# Synbiotics modulate gut microbiota and reduce enteritis and ventilator-associated pneumonia in patients with sepsis: a randomized controlled trial

**DOI:** 10.1186/s13054-018-2167-x

**Published:** 2018-09-27

**Authors:** Kentaro Shimizu, Tomoki Yamada, Hiroshi Ogura, Tomoyoshi Mohri, Takeyuki Kiguchi, Satoshi Fujimi, Takashi Asahara, Tomomi Yamada, Masahiro Ojima, Mitsunori Ikeda, Takeshi Shimazu

**Affiliations:** 1 Department of Traumatology and Acute Critical Medicine, Osaka University Graduate School of Medicine, 2-15 Yamadaoka, Suita-city, Osaka, 565-0871 Japan; 2 Department of Trauma, Critical care and Emergency medicine, Osaka General Medical Center, Osaka, Japan; 3 Yakult Central Institute, Kunitachi-shi, Tokyo Japan; 4 Department of Medical Innovation, Osaka University Hospital, Suita, Osaka, Japan

**Keywords:** Sepsis, Synbiotics, Probiotics, Gut, Microbiota, Short-chain fatty acids, Diarrhea, Ventilator-associated pneumonia

## Abstract

**Background:**

Commensal microbiota deteriorate in critically ill patients. The preventive effects of probiotic/synbiotic therapy on microbiota and septic complications have not been thoroughly clarified in patients with sepsis. The objective of this study was to evaluate whether synbiotics have effects on gut microbiota and reduce complications in mechanically ventilated patients with sepsis.

**Methods:**

Sepsis patients who were mechanically ventilated in the intensive care unit (ICU) were included in this randomized controlled study. Patients receiving daily synbiotics (*Bifidobacterium breve* strain Yakult, *Lactobacillus casei* strain Shirota, and galactooligosaccharides) initiated within 3 days after admission (the Synbiotics group) were compared with patients who did not receive synbiotics (the No-Synbiotics group). The primary outcome was infectious complications including enteritis, ventilator-associated pneumonia (VAP), and bacteremia within 4 weeks from admission. The secondary outcomes included mortality within 4 weeks, fecal bacterial counts, and organic acid concentration. Enteritis was defined as the acute onset of continuous liquid stools for more than 12 h.

**Results:**

Seventy-two patients completed this trial; 35 patients received synbiotics and 37 patients did not receive synbiotics. The incidence of enteritis was significantly lower in the Synbiotics than the No-Synbiotics group (6.3% vs. 27.0%; *p* < 0.05). The incidence of VAP was also significantly lower in the Synbiotics than the No-Synbiotics group (14.3% vs. 48.6%; *p* < 0.05). The incidence of bacteremia and mortality did not differ significantly between the two groups. In the analysis of fecal bacteria, the number of *Bifidobacterium* and *Lactobacillus* in the Synbiotics group was significantly higher than that in the No-Synbiotics group. In the analysis of fecal organic acids, total organic acid concentration, especially the amounts of acetate, were significantly greater in the Synbiotics group than in the No-Synbiotics group at the first week (*p* < 0.05).

**Conclusions:**

Prophylactic synbiotics could modulate the gut microbiota and environment and may have preventive effects on the incidence of enteritis and VAP in patients with sepsis.

**Trial registration:**

UMIN, R000007633. Registered on 29 September 2011.

## Background

The gut is a critical target organ for many kinds of stress such as trauma, burn, shock, bleeding, and infection [1]. A severe insult to the gut is believed to promote infectious complications and multiple organ dysfunction syndrome, the causes of which include deterioration of the intestinal epithelium, the immune system, and commensal bacteria [2]. Normal gut microbiota have an important role in metabolism, nutrition, and protection against pathogens [3]. Disruption of the gut microbiota, or “dysbiosis”, could lead to many diseases such as infection, inflammatory bowel disease, metabolic syndrome, and cancer. In critically ill patients, the gut microbiota is altered significantly especially with regard to the number of obligate anaerobes, which are the dominant bacteria and are associated with infectious complications and mortality [4].

Probiotics are defined by the Food and Agriculture Organization (FAO)/World Health organization (WHO) as live microorganisms which, when administered in adequate amounts, confer a health benefit on the host and are widely used as a live microbial food supplement that can improve the intestinal microbial balance [5]. *Lactobacillus* and *Bifidobacterium* are popular probiotics. Probiotics have been shown to exert health benefits, such as an anticolon cancer effect and resistance to enteric pathogens, immune system modulation, allergy, inflammatory bowel diseases, and necrotizing enterocolitis [6]. Prebiotics are defined as a nondigestible food ingredient that beneficially affects the host by selectively stimulating the growth and/or activity of one or a limited number of bacterial species in the colon [7]. Synbiotics consist of probiotics and prebiotics. There are several reports on the effects of probiotics and synbiotics in sepsis patients. Shimizu et al. [8] reported that 29 patients with systemic inflammatory response syndrome who received *Bifidobacterium breve* strain Yakult and *Lactobacillus casei* strain Shirota had fewer complications of diarrhea during their intensive care unit (ICU) stay. For critically ill patients, probiotics and synbiotics reduced infectious complications, especially for elective surgery and trauma [9]. There are few reports on the effects of probiotics and synbiotics in sepsis patients. Therefore, the objective of this study was to evaluate whether synbiotics maintain the microbiota and reduce infectious complications in mechanically ventilated patients with sepsis.

## Methods

### Patients

Patients who were more than 16 years old and were placed on a ventilator within 3 days after admission to the ICU, and who were diagnosed as having sepsis in the Department of Traumatology and Acute Critical Medicine, Osaka University Medical School, and Osaka General Medical Center during the period November 2011 to September 2016, were eligible for enrollment in this randomized controlled study [10]. Patients were randomly assigned in a 1:1 ratio to treatment groups using permutation blocks (*n* = 4 per block). The allocation sequence was generated by the corresponding author. The sequence was kept secure from all study personnel responsible for screening and recruiting patients. This was a single-blind study in which the participants were blinded. The patients who received synbiotics were assigned to the Synbiotics group, and the patients who received no synbiotics were assigned to the No-Synbiotics group. Patients were excluded if they were receiving other probiotics or were expected to be discharged or transferred out of the ICU within 3 days after admission.

Sample size calculations assumed a 30% incidence of infectious complications in the control arm based on existing published data from this ICU, a 10% incidence by the intervention, and a dropout rate of 10%. We calculated that approximately 136 patients should be enrolled to achieve a statistical power of 80% with a two-sided significance level of at least 0.05. We thus registered 150 patients as a target.

### Interventions

The probiotics used were Yakult BL Seichoyaku (Yakult Honsha, Tokyo, Japan), which contained 1 × 10^8^ living bacteria of the *B. breve* strain Yakult/g and 1 × 10^8^ living bacteria of the *L. casei* strain Shirota/g. The prebiotics used were galactooligosaccharides (Oligomate S-HP, Yakult Honsha). Yakult BL Seichoyaku (3 g/day) and galactooligosaccharides (10 g/day) were administered as synbiotic therapy. The synbiotics in the Synbiotics group were initiated within 3 days after admission when enteral nutrition was started via nasal tube and were continued until oral intake was initiated. Enteral nutrition using a standard polymeric diet Glucerna®-Ex (Abbott Japan Co. Ltd., Tokyo, Japan; 1 kcal/mL; 51:17:32 ratio of carbohydrate, protein, and fat; 370 mOsm/L; fiber 1.4 g/100 mL formula) was initiated as soon as possible through a nasogastric tube at 20 mL/h and advanced by 20 mL/h/day to the calorie goal. During the study period, we used 25–30 kcal/kg ideal body weight per day as the calorie goal. If infections occurred, patients were initially treated empirically for the underlying clinical syndrome and then according to the results of antibiotic susceptibility testing of the bacterial isolate causing the infection. Antibiotics were administered under the same policy during the entire study period. This study was approved by the institutional review board of Osaka University. Informed consent was obtained from the family of each patient. The clinical trial registry number is UMIN R000007633.

### Determination of fecal microbiota counts

Fecal samples were acquired from the subjects by swabs of the rectum. Samples were collected weekly. Samples with a cotton applicator were put into test tubes containing 1 ml RNA *later*® (Ambion, Inc., Austin, TX, USA), an RNA stabilization solution, prior to bacteriological analysis, and then the samples were incubated for 5 min at room temperature. Samples with a cotton applicator were put into test tubes prior to fecal organic acid analysis. All samples were stored at −20 °C until analysis. RNA was isolated using the method described elsewhere [11, 12]. Finally, the nucleic acid fraction was suspended in 1 mL nuclease-free water. The microbiota composition was analyzed using the Yakult Intestinal Flora-SCAN (YIF-SCAN®) version of a 16S and 23S rRNA-targeted reverse-transcription quantitative polymerase chain reaction (RT-qPCR) system. A standard curve was generated with RT-qPCR using the threshold cycle (C_T_) value, i.e., the cycle number when the threshold fluorescence was reached, and the corresponding cell count was determined microscopically with 4,6-diamidino-2-phenylindole (Vector Laboratories, Burlingame, CA) staining for a dilution series of the standard strains as described elsewhere [12]. To determine the types of bacteria present in the samples, three serial dilutions of an extracted RNA sample were used for RT-qPCR, and the C_T_ values in the linear range of the assay were applied to the standard curve to obtain the corresponding bacterial cell counts in each nucleic acid sample. These data were then used to determine the number of bacteria per sample. The specificity of the RT-qPCR assay using group-, genus-, or species-specific primers was determined as described previously [11, 12]. The quantitative analyses of *L. casei* strain Shirota [13] and *B. breve* strain Yakult [14] have been described previously.

### Determination of fecal organic acid concentrations

A portion of the feces was isolated, weighed, mixed with 0.15 M perchloric acid at a fourfold volume, and stored at 4 °C for 12 h. The mixture was centrifuged at 4 °C at 20,400×*g* for 10 min, and the supernatant was filtrated with a 0.45-μm membrane filter (Millipore Japan Ltd., Tokyo, Japan) and sterilized. The sample was analyzed for organic acids by high-performance liquid chromatography, which was performed with a Waters system (Waters 432 Conductivity Detector; Waters Co., Milford, MA) equipped with two columns (Shodex RS pack KC-811; Showa Denko Co. Ltd., Tokyo, Japan). The concentrations of organic acids were calculated with the use of external standards, and the reproducibility and stability of these measurements have been shown previously [15].

### Surveillance and definition of infection

Body temperature was measured continuously. Surveillance cultures from the urine, blood, and sputum were routinely performed once a week for each patient. In cases of suspected infection, laboratory tests, chest x-rays, and computed tomography scans were performed when necessary. Bacterial infection was diagnosed based on the Centers for Disease Control definitions during the 28 days after admission [16]. Enteritis was defined as the acute onset of continuous liquid stools for more than 12 h. Ventilator-associated pneumonia (VAP) refers to pneumonia that arises more than 48–72 h after endotracheal intubation [17]. Bacteremia was defined as a positive blood culture after the first 3 days. Pneumonia-free days were defined as the period until patients were first diagnosed as having VAP during the 28 days after admission.

The primary outcome was enteritis, VAP, and bacteremia within 4 weeks from admission. The secondary outcomes included mortality within 4 weeks, fecal bacterial counts, and organic acid concentration.

### Statistical analysis

For fecal microbiota and organic acid analysis, results are expressed as mean ± standard error (SE) values. For the statistical calculation of fecal bacterial count and organic acid concentration, a value of half of the detection limit was assigned in case the count or concentration was below the detection limit. To account for dependencies in repeatedly measured observations within a subject, a linear mixed-effect model was used with group, week, and interaction of group and week. Multiple comparisons were adjusted by Bonferroni’s correction. The Acute Physiology and Chronic Health Evaluation (APACHE) II score was assessed on admission, and the initial values were used as covariates. For the incidence of infectious complications, Cox proportional hazards models were used to estimate occurrences during the first 28 days as outcomes with adjustment for APACHE II score and sex. The cumulative incidence of infectious complications was evaluated by log-rank test. A significance level of a two-sided *p* < 0.05 was used for statistical inferences. Statistical analyses were performed using SPSS (version 22, SPSS, Chicago, IL, USA), and data are presented using GraphPad Prism, version 6.04 (GraphPad Software, La Jolla, CA, USA).

## Results

**Fig. 1:**
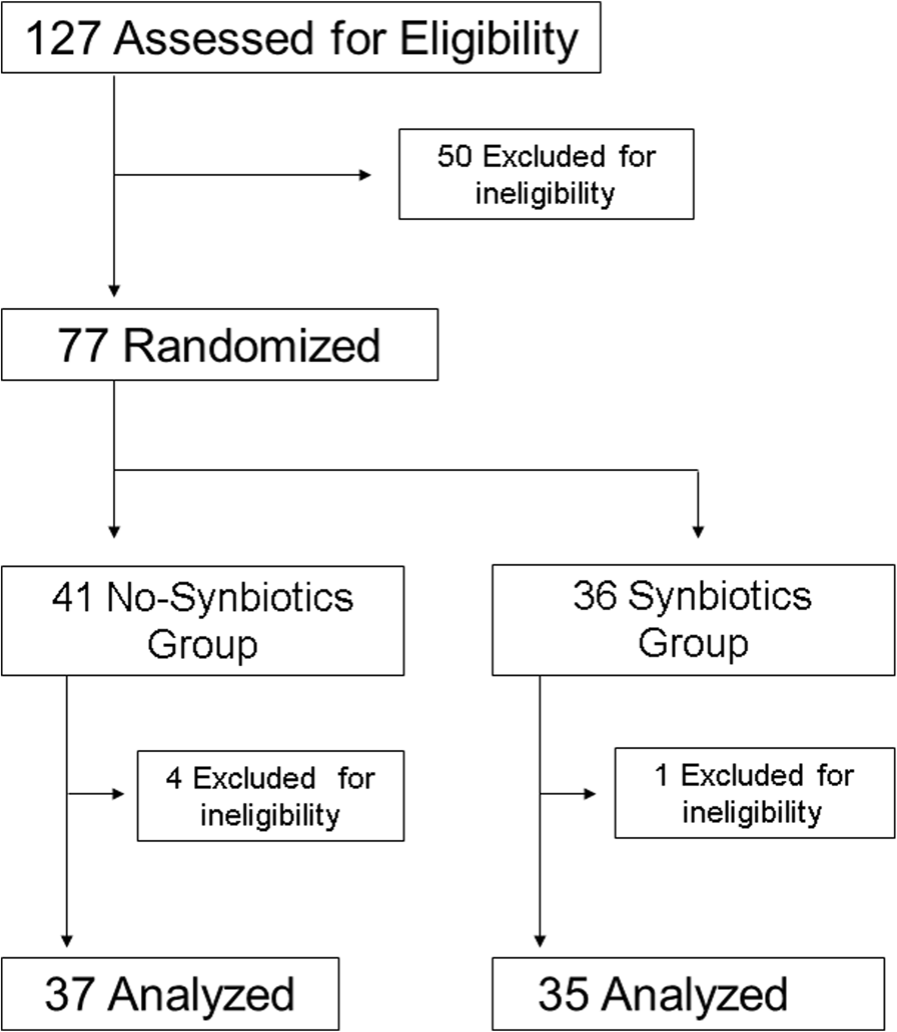
Study participants and flow chart

Of the 127 patients assessed, 50 patients were excluded because other probiotics were used or the patients were too severely ill to survive. Thus, 77 patients were randomized, of whom 72 patients completed this trial, with 35 patients receiving synbiotics and 37 patients not receiving synbiotics (Fig. 1). Reasons for ineligibility included other probiotics (*B. bifidum*, *Clostridium butyricum*) being used after randomization. No adverse events occurred in any of the patients. Patient characteristics are listed in Table 1. The two groups did not differ significantly in terms of age, sex, APACHE II score on admission, comorbidities, or the cause of sepsis. The principal diseases in sepsis were respiratory, intra-abdominal, and skin/soft tissue infection. The median levels of blood lactate on admission were 33 mg/dL in the Synbiotics group and 22 mg/dl in the No-Synbiotics group (*p* > 0.05). In the Synbiotics group, synbiotics were used for a median of 20 (interquartile range (IQR) 10–36) days.

**Table 1:** Patient characteristics

	No-Synbiotics	Synbiotics	*p* value
Patients (*n* = 72)	37	35	
Age (years), median (IQR)	74 (64–81)	74 (64–82)	0.73
Gender (male), *n* (%)	22 (59)	25 (71)	0.29
APACHE II score, median (IQR)	20 (14–26)	19 (14–24)	0.47
Comorbidities, *n* (%)			
Heart disease	15 (41)	14 (40)	0.96
Diabetes mellitus	14 (38)	15 (43)	0.66
Immunocompromised disease	8 (22)	11 (31)	0.34
Malignancy	5 (14)	7 (20)	0.46
Chronic renal disease	4 (11)	5 (14)	0.66
Pulmonary disease	3 (8)	3 (9)	0.94
Origin of sepsis, *n* (%)			
Respiratory	16 (43)	19 (54)	
Intra-abdominal	4 (11)	5 (14)	
Skin/soft tissue	4 (11)	5 (14)	
Urinary tract infection	6 (16)	1 (3)	
CNS	0 (0)	2 (6)	
Others	7 (19)	3 (9)	
Blood lactate, median (IQR)	22 (14–60)	33 (20–51)	0.49

*APACHE* Acute Physiology and Chronic Health Evaluation, *CNS* central nervous system, *IQR* interquartile range

In the analysis of fecal microbiota, the number of total bacteria in the Synbiotics group increased significantly compared with that in the No-Synbiotics group by a linear mixed-effect model (*p* < 0.05). The numbers of *Bifidobaterium* and *Lactobacillus* and *Atopobium* clusters in the Synbiotics group were especially significantly higher than those in the No-Synbiotics group (Table 2). In the analysis of fecal organic acids, the total organic acid concentration, especially the amounts of acetate, was significantly greater in the Synbiotics group than in the No-Synbiotics group at the first week by Bonferroni’s multiple comparison (*p* < 0.05; Table 3). The statistical significance did not change after adjustment for APACHE II score on admission and the initial values as covariates.

**Table 2:** Serial changes in fecal microbiota in the Synbiotics and No-Synbiotics groups

	Initial		1 week		2 weeks		*p* value		
	No-Synbiotics	Synbiotics	No-Synbiotics	Synbiotics	No-Synbiotics	Synbiotics	Group	Week	Group×week
Total bacteria	8.1 ± 0.4	6.8 ± 0.4	7.7 ± 0.4	8.9 ± 0.4	8.3 ± 0.4	8.6 ± 0.4	0.190	< 0.05	< 0.05
*Clostridium coccoides* group	5.1 ± 1.0	4.4 ± 1.0	3.0 ± 1.0	5.5 ± 1.0	5.6 ± 1.1	6.0 ± 1.1	0.946	0.182	0.301
*Clostridium leptum* subgroup	6.7 ± 1.0	4.9 ± 1.0	5.5 ± 1.0	6.3 ± 1.0	5.8 ± 1.0	6.0 ± 1.1	0.282	0.727	0.136
*Bacteroides fragilis* group	5.6 ± 1.1	4.5 ± 1.1	4.8 ± 1.1	4.4 ± 1.1	7.3 ± 1.2	5.9 ± 1.2	0.425	0.086	0.992
*Bifidobacterium*	5.6 ± 1.0	4.1 ± 1.0	3.1 ± 1.0	6.2 ± 1.0	3 ± 1.1	6.8 ± 1.1	0.358	0.933	< 0.05
*Atopobium* cluster	6.4 ± 0.9	4.7 ± 0.9	5.2 ± 0.9	5.9 ± 0.9	4.8 ± 1.0	5.8 ± 1.0	0.133	0.897	< 0.05
*Prevotella*	1.9 ± 0.8	4.1 ± 0.9	0.9 ± 0.9	2.3 ± 0.9	1.3 ± 0.9	1.7 ± 0.9	0.092	0.061	0.299
*Clostridium perfringens*	1.9 ± 0.4	1.9 ± 0.5	2.1 ± 0.4	1.5 ± 0.5	1.7 ± 0.5	1.5 ± 0.5	0.790	0.272	0.660
Total *Lactobacillus*	4.5 ± 0.6	3.7 ± 0.6	4.1 ± 0.6	6.2 ± 0.6	3.9 ± 0.7	6.3 ± 0.7^a^	0.689	0.069	< 0.05
*L. gasseri* subgroup	3.5 ± 0.5	3.0 ± 0.6	2.5 ± 0.6	4 ± 0.6	2.3 ± 0.6	3.9 ± 0.6	0.781	0.791	0.060
*L. brevis*	1.4 ± 0.2	1.1 ± 0.3	1.3 ± 0.3	1.1 ± 0.3	1.3 ± 0.3	1.1 ± 0.3	–	–	–
*L. casei* subgroup	2.9 ± 0.5	2.7 ± 0.5	2.7 ± 0.5	5.6 ± 0.5^a^	2.7 ± 0.5	5.5 ± 0.6^a^	0.631	< 0.05	< 0.05
*L. fermentum*	3.7 ± 0.6	2.5 ± 0.6	3.3 ± 0.6	3.1 ± 0.6	3.1 ± 0.6	3.6 ± 0.6	0.081	0.505	0.071
*L. plantarum* subgroup	1.4 ± 0.2	1.2 ± 0.2	1.5 ± 0.2	1.4 ± 0.2	1.3 ± 0.2	1.2 ± 0.2	–	–	–
*L. reuteri* subgroup	3.2 ± 0.6	1.9 ± 0.6	2.9 ± 0.6	3.0 ± 0.6	3.0 ± 0.7	3.2 ± 0.7	0.169	0.315	0.170
*L. ruminis* subgroup	2.7 ± 0.7	2.4 ± 0.7	2.9 ± 0.7	2.9 ± 0.7	2.6 ± 0.7	3.0 ± 0.7	0.742	0.597	0.441
*L. sakei* subgroup	1.2 ± 0.2	1.5 ± 0.3	1.4 ± 0.3	2.4 ± 0.3	1.2 ± 0.3	1.4 ± 0.3	0.100	0.888	0.818
*Enterobacteriaceae*	5.1 ± 0.7	4.8 ± 0.7	4.9 ± 0.7	4.5 ± 0.7	5.7 ± 0.8	5.3 ± 0.8	0.751	0.411	0.922
*Enterococcus*	4.4 ± 0.6	3.9 ± 0.7	5.3 ± 0.7	7.3 ± 0.7	6.5 ± 0.7	6.1 ± 0.7	0.751	< 0.05	0.886
*Staphylococcus*	3.4 ± 0.6	3.0 ± 0.6	4.2 ± 0.6	4.7 ± 0.6	3.3 ± 0.6	3.7 ± 0.7	0.832	0.546	0.466
*Pseudomonas*	1.5 ± 0.5	1.5 ± 0.6	2.3 ± 0.6	2.5 ± 0.6	3.9 ± 0.6	2.2 ± 0.6	0.464	< 0.05	0.089
*Lactobacillus casei* strain Shirota	3.1 ± 0.3	3.4 ± 0.5	2.9 ± 0.3	4.3 ± 0.5	2.5 ± 0.3	4.8 ± 0.6^a^	0.531	0.359	< 0.05
*Bifidobacterium breve* strain Yakult	3.3 ± 0.3	2.9 ± 0.6	2.5 ± 0.3	4.9 ± 0.6†	2.5 ± 0.3	4.8 ± 0.6^a^	0.894	0.253	< 0.05

Values are mean ± SE (log_10_ cells/g of feces)

*p* value by linear mixed-effects model

^a^Statistical significance between groups determined with Bonferroni’s correction

**Table 3:** Serial changes in fecal organic acids in the Synbiotics and No-Synbiotics groups

	Initial		1 week		2 weeks		*p* value		
	No-Synbiotics	Synbiotics	No-Synbiotics	Synbiotics	No-Synbiotics	Synbiotics	Group	Week	Group×week
Total organic acids	44.2 ± 11.5	49.1 ± 12.1	46.2 ± 12	116.6 ± 12.1^a^	54.8 ± 12.6	71.4 ± 12.6	0.169	0.161	0.664
Succinic acid	0.1 ± 12.4	0.8 ± 12.4	3.4 ± 13	1.4 ± 12.4	49.1 ± 13.7	1.1 ± 13.7	0.335	0.078	0.091
Lactic acid	2.6 ± 5.0	4.4 ± 5.2	1.3 ± 5.1	16.9 ± 5.2	7.4 ± 5.3	13.1 ± 5.4	0.287	0.089	0.565
Formic acid	5.5 ± 4.4	13.4 ± 4.6	6.5 ± 4.5	10.4 ± 4.6	4.4 ± 4.7	6.9 ± 4.7	0.370	0.391	0.465
Acetic acid	33.9 ± 7.6	22.5 ± 8.0	25.8 ± 7.9	61.0 ± 8.0^a^	23.5 ± 8.2	41.6 ± 8.3	0.901	0.506	0.058
Propionic acid	1.3 ± 2.6	2.5 ± 2.7	5.8 ± 2.7	9.1 ± 2.7	4.7 ± 2.9	3.2 ± 2.9	0.491	0.414	0.625
Butyric acid	0.3 ± 3.4	4.8 ± 3.6	1.8 ± 3.5	17.1 ± 3.6	0.2 ± 3.6	3.9 ± 3.6	0.642	0.731	0.722

Values are mean ± SE (μmol/g feces)

*p* value by linear mixed effects model

^a^Statistical significance between groups determined with Bonferroni’s correction

In the analysis of complications, the incidence of infectious complications during the 28 days after admission was significantly lower in the Synbiotics group versus the No-Synbiotics group (28.6% vs. 67.6%; *p* < 0.05; Table 4).The incidence of enteritis during the 28 days after admission was significantly lower in the Synbiotics group versus the No-Synbiotics group (6.3% vs. 27.0%; *p* < 0.05). The incidence of VAP during the 28 days after admission was also significantly lower in the Synbiotics group versus the No-Synbiotics group (14.3% vs. 48.6%; *p* < 0.05). The number of ventilator-free days at day 28 did not differ significantly between the No-Synbiotics group (median 7, IQR 4.5–19.5 days) and the Synbiotics group (median 14, IQR 0–21 days). There were no significant differences in the incidence of bacteremia (14.3% vs. 13.5%) or mortality (8.6% vs. 10.8%) due to multiple organ dysfunction syndrome between the two groups during the 28 days after admission (Synbiotics group vs. No-Synbiotics group). All antibiotics were administered intravenously. There were no significant differences in the duration of antibiotic administration (mean 13, IQR 8–24 days vs. 18 (10–28) days) or in the kinds of antibiotics used (4 (3–6) vs. 4 (2–6)) between the Synbiotics group and the No-Synbiotics group. Carbapenem antibiotics were used in 74.3% of the patients in the Synbiotics group and in 75.7% of patients in the No-Synbiotics group.

**Table 4:** Complications and antibiotics

	No-Synbiotics (*n* = 37)	Synbiotics (*n* = 35)	*p* value
Infectious complications, *n* (%)	25 (67.6)	10 (28.6)	< 0.05
Enteritis, *n* (%)	10 (27.0)	2 (6.3)	< 0.05
Onset day, median (IQR)	15 (9–23)	9.5 (6–13)	0.41
Ventilator-associated pneumonia, *n* (%)	18 (48.6)	5 (14.3)	< 0.05
Onset day, median (IQR)	8 (5–21)	8 (5–13)	0.68
Bacteremia, *n* (%)	5 (13.5)	5 (14.3)	0.92
Mortality, *n* (%)	4 (10.8)	3 (8.6)	0.84
ICU stay, median (IQR)	28 (17–45)	23 (13–43)	0.85
Antibiotics, *n* (%)			
Carbapenem	28 (75.7)	26 (74.3)	0.90
Ampicillin/sulbactam	18 (48.6)	17 (48.6)	0.99
Cephalosporin	13 (35.1)	12 (34.3)	0.95
Vancomycin	11 (29.7)	11 (31.4)	0.87
Quinolone	8 (21.6)	12 (34.3)	0.22
Penicillin class	5 (13.5)	6 (17.1)	0.66
Antibiotics duration (days), median (IQR)	18 (10–28)	13 (8–24)	0.29

**Fig. 2:**
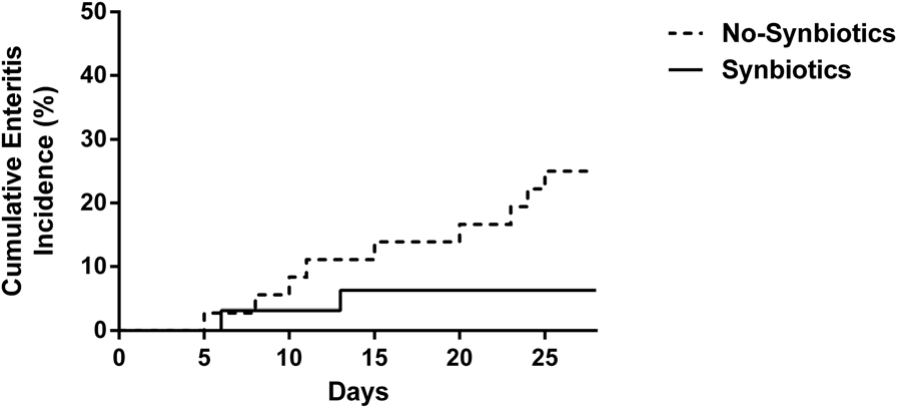
The cumulative incidences of enteritis were significantly lower in the Synbiotics group than in the No-Synbiotics group by log-rank test (*p* < 0.05)

**Fig. 3:**
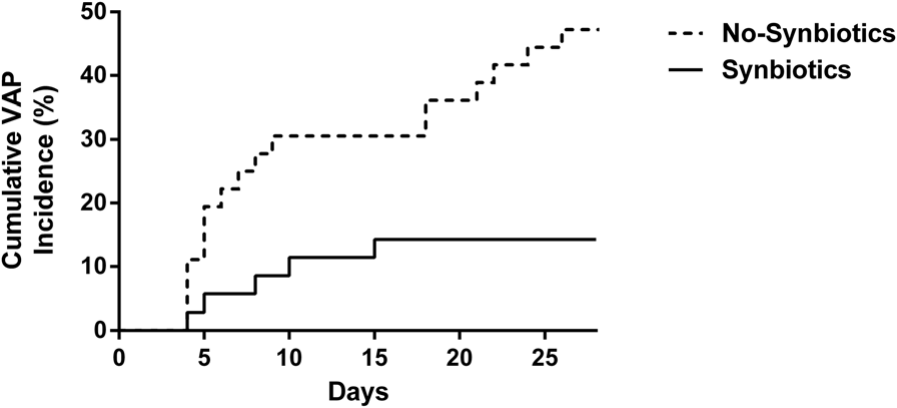
The cumulative incidences of ventilator-associated pneumonia (VAP) were significantly lower in the Synbiotics group than in the No-Synbiotics group by log-rank test (*p* < 0.05)

*ABPC/SBT* ampicillin sulbactam, *IQR* interquartile range,

In a Cox proportional hazards model, the hazard ratios for the occurrence of enteritis during the first 28 days in the Synbiotics group compared with the No-Synbiotics group were 0.18 (95% confidence interval (CI) 0.026 to 0.760; *p* = 0.036) and 0.15 (95% CI 0.021 to 0.685; *p* = 0.012), respectively, when adjusted for sex and APACHE II score. The hazard ratios for the occurrence of VAP during the first 28 days in the Synbiotics group versus the No-Synbiotics group were 0.19 (95% CI 0.057 to 0.584; *p* = 0.005) and 0.20 (95% CI 0.057 to 616; *p* = 0.004), respectively, when adjusted for sex and APACHE II score. The cumulative incidences of enteritis and VAP were significantly lower in the Synbiotics group than those in the No-Synbiotics group by log-rank test (*p* < 0.05; Figs. 2 and 3).

## Discussion

This study was a randomized controlled study to determine whether prophylactic synbiotics maintained gut microbiota and prevented the occurrence of enteritis and VAP in patients with sepsis. Sepsis treatment including massive infusion, inotropic therapy, antibiotics, and other therapies, can affect the human gut microbiota following sepsis. Shimizu et al. reported that the number of obligate anaerobes, especially *Bifidobacterium* and *Bacteroides*, were decreased and continued in patients with systemic inflammatory response syndrome (SIRS) [18, 19]. The number of obligate anaerobes were around 10 (log_10_ colony-forming units (CFUs)/g of feces) on average in normal people and postoperative patients [20, 21]. In the present study, the number of total bacteria tended to decrease to about 6 (log_10_ CFUs/g of feces) on average in the No-Synbiotics group, which was much less than that in normal people and postoperative patients. The numbers of pathogenic bacteria such as *Enterococcus* and *Pseudomonas* increased significantly within 1 week in both groups (*p* < 0.05), but they did not reach statistical significance with the interaction of group and week. The numbers of pathogenic bacteria, such as total facultative anaerobes, and also those of total obligate anaerobes were the significant prognostic factors in patients with SIRS [4]. These findings suggest that sepsis influenced the microbiota of the patients and might be related to the occurrence of subsequent complications.

Synbiotics, as a combination of probiotics and prebiotics, have been reported to promote immunity against severe injuries such as trauma and infection. Although the mechanisms of probiotics have not yet been clarified, one of the important factors is microorganism-host crosstalk such as microorganism-associated molecular patterns (MAMPs) of probiotics and pattern recognition receptors (PRRs) of the gastrointestinal mucosa [22]. The most well-known PRRs are Toll-like receptors (TLRs). MAMPs consist of flagellin, lipopolysaccharide, peptidoglycan, and other factors. For example, flagellins of the probiotic *Escherichia. coli* Nissle 1917 were shown to induce beta-defensin via TLR5 [23]. Asahara et al. reported that intraluminal acetate produced by *B. breve* strain Yakult could inhibit the toxin in a mouse model of toxin-producing *E. coli* infection [24]. The *L. casei* strain Shirota was effective against multidrug resistant *Salmonella enterica* serovar Typhimurium DT104 infection in a 5-FU treated mouse model [25]. In the present report, administered *B. breve* strain Yakult and *L. casei* strain Shirota might inhibit pathogenic bacteria and toxins through signal interaction and prevent septic complications. Also, the number of total bacteria was significantly higher in the Synbiotics group. The numbers of *Bifidobacterium* and total *Lactobacillus* in the Synbiotics group were especially increased over those in the No-Synbiotics group. Looking at total *Lactobacillus*, the *L. gasseri* subgroup and *L. fermentum* tended to increase more in the Synbiotics group than in the No-Synbiotics group. *L. gasseri* has a role in vaginal homeostasis and *Helicobacter pylori* infection and improvement of diarrhea [26]. *L. fermentum* is reported to enhance the immunologic response of influenza vaccination [27]. These findings suggest that synbiotics not only increase the number of administered bacteria but also increase their genus groups and other microbiota, which could lead to the maintenance of gut microbiota. In previous studies, synbiotics could maintain gut microbiota following SIRS [8] and major surgeries [21]. Synbiotics could have a supplemental effect to increase the number of microbiota.

Short-chain fatty acids (SCFAs) consist of acetic, propionic, and butyric acids with 2–4 carbon atoms. Anaerobic metabolism of peptides and proteins by the microbiota produces SCFAs that all have important functions in host physiology. SCFAs are utilized mainly by intestinal epithelial cells as energy substrates, and some are absorbed into the portal flow to the liver and utilized as systemic energy sources [28]. SCFAs bind to the G-protein-coupled-receptor 43 (GPR43). Maslowski et al. reported that GPR43-deficient mice showed exacerbation of inflammation in models of colitis, arthritis, and asthma [29]. Asahara et al. reported that intraluminal acetate produced by synbiotics could inhibit the toxin in a mouse model of toxin-producing *E. coli* infection [24]. In the present report, the synbiotic-treated group had significantly maintained gut microbiota and organic acids, especially acetate. Increased levels of acetate might attenuate inflammation to reduce septic complications. Butyric acid in the feces of the patients decreased in both groups from the normal values (16.6 ± 6.7 μmol/g (mean ± SD)) described in our previous paper [18], which could be due to the decreased numbers of bacteria and lactate levels. The differences in the values did not reach statistical significance between the groups in the present study.

Regarding diarrhea in the ICU, Bleichner et al. reported that in 128 ICU patients the number of days with diarrhea was reduced in patients treated with *Saccharomyces boulardii* [30]. In our previous study of SIRS, the patients treated with synbiotics had a significantly reduced incidence of diarrhea compared with the controls [8]. In the present research, synbiotics showed beneficial effects against complications of enteritis in the patients with sepsis. Prophylactic synbiotics could maintain gut microbiota and reduce the incidence of enteritis. Further study is needed to determine the mechanisms of the prevention of diarrhea.

There are several reports on the effectiveness of probiotics and synbiotics on the incidence of VAP [31]. Morrow et al. [32] reported that the incidence of VAP in patients treated with *L. rhamnosus* GG was significantly lower than that in the controls (19.1% vs. 40.0%) in 138 ICU patients. Also, probiotic administration significantly reduced oropharyngeal and gastric colonization of pathogenic species. Fukuda et al. reported that *Bifidobacteria* continue to generate acetate through ATP-binding cassette-type carbohydrate transporter and prevent translocation in a mouse model [33]. Also, intraluminal acetate could increase the level of tight junction proteins including claudin-1, occludin, and ZO-1, which could prevent bacterial translocation in a mouse model of *Acinetobacter baumannii* infection [34]. In our report, the synbiotic-treated group had significantly maintained gut microbiota and organic acids, especially acetate. Increased levels of acetate and lactate might inhibit intraluminal toxins and maintain tight junctions. These changes indicated that synbiotic treatment could have beneficial effects on microbiota and reduce the development of VAP. However, other clinical reports showed no significant difference in the occurrence of VAP in the ICU [35]. One of the reasons is the difference in administered bacteria. In a mouse model, the antitoxic effects and organic acid concentration of probiotics such as *Bifidobacterium* and *Lactobacillus* are different with species [24, 25, 33]. In a rabbit infective endocarditis model, the incidence of infection was different with *Lactobacillus* species [36]. Besselink et al. [37] reported that mortality rates with six kinds of bacteria were significantly higher than those without these bacteria in the PROPATRIA study (16% vs. 6%). However, the incidence of infectious complications showed no significant differences and, in addition, the study has been criticized from multiple perspectives [38]. The effects of synbiotics for gut microbiota might be different with different species or combinations of bacteria and different diseases. Further analysis is needed to determine the appropriate probiotic species for preventing VAP and to elucidate the underlying mechanism of synbiotic treatment.

This study has some limitations. There is a source of potential bias in that gut microbiomes are different with different backgrounds such as geography, ethnicity, and lifestyle [39]. Thus, innate immunity via gut microbiota could be different against pathogenic bacteria. The modulation of gut microbiota by synbiotics might be different with these backgrounds. Second, there is limited generalizability. Because patients were collected through a tertiary center after being transferred directly from the emergency medical system in a limited area, the studied patients do not represent the national population, and the ethnicity of the subjects was only Asian. Therefore, caution is required in applying the findings to a larger worldwide population. Third, this is a quantitative research study of the main subset of microbiota, and changes in other whole bacteria were not evaluated. The bacteria influenced by synbiotics in the gut microbiome could be a target for further study. Fourth, the number of patients was lower than expected because of the small number of sepsis patients requiring mechanical ventilation with early enteral nutrition. Additional multicenter studies are needed to solve this problem. Fifth, cost-effectiveness analysis using measures such as the number needed to treat could be needed for further research to apply synbiotics clinically.

## Conclusions

The administration of synbiotics increased the levels of beneficial bacteria and SCFAs. The beneficial alterations of gut microbiota and environment may decrease the incidence of enteritis and VAP in patients with sepsis. Further research is needed to investigate the effects of synbiotic treatment.

## Abbreviations

APACHE: Acute Physiology and Chronic Health Evaluation
CFU: Colony-forming unit
CI: Confidence interval
C_T_: Threshold cycle
GPR43: G-protein-coupled-receptor 43
ICU: Intensive care unit
IQR: Interquartile range
MAMP: Microorganism-associated molecular pattern
PRR: Pattern recognition receptor
RT-qPCR: Reverse-transcription quantitative polymerase chain reaction
SCFA: Short-chain fatty acid
SIRS: Systemic inflammatory response syndrome
TLR: Toll-like receptor
VAP: Ventilator-associated pneumonia

## References

[1] MacFie J, O’Boyle C, Mitchell CJ, Buckley PM, Johnstone D, Sudworth P (1999). Gut origin of sepsis: a prospective study investigating associations between bacterial translocation, gastric microflora, and septic morbidity. Gut.

[2] Clark JA, Coopersmith CM (2007). Intestinal crosstalk: a new paradigm for understanding the gut as the “motor” of critical illness. Shock.

[3] Guarner F, Malagelada JR (2003). Gut flora in health and disease. Lancet.

[4] Shimizu K, Ogura H, Hamasaki T, Goto M, Tasaki O, Asahara T (2011). Altered gut flora are associated with septic complications and death in critically ill patients with systemic inflammatory response syndrome. Dig Dis Sci.

[5] FAO/WHO (2001). Report of a joint FAO/WHO expert consultation on evaluation of health and nutritional properties of probiotics in food including powder milk with live lactic acid bacteria.

[6] Nagpal R, Kumar A, Kumar M, Behare PV, Jain S, Yadav H (2012). Probiotics, their health benefits and applications for developing healthier foods: a review. FEMS Microbiol Lett.

[7] Gibson GR, Roberfroid MB (1995). Dietary modulation of the human colonic microbiota: introducing the concept of prebiotics. J Nutr.

[8] Shimizu K, Ogura H, Goto M, Asahara T, Nomoto K, Morotomi M (2009). Synbiotics decrease the incidence of septic complications in patients with severe SIRS: a preliminary report. Dig Dis Sci.

[9] Shimizu K, Ogura H, Asahara T, Nomoto K, Morotomi M, Tasaki O (2013). Probiotic/synbiotic therapy for treating critically ill patients from a gut microbiota perspective. Dig Dis Sci.

[10] Bone RC, Balk RA, Cerra FB, Dellinger RP, Fein AM, Knaus WA (1992). Definitions for sepsis and organ failure and guidelines for the use of innovative therapies in sepsis. The ACCP/SCCM consensus conference committee. American College of Chest Physicians/Society of Critical Care Medicine. Chest.

[11] Matsuda K, Tsuji H, Asahara T, Kado Y, Nomoto K (2007). Sensitive quantitative detection of commensal bacteria by rRNA-targeted reverse transcription-PCR. Appl Environ Microbiol.

[12] Matsuda K, Tsuji H, Asahara T, Matsumoto K, Takada T, Nomoto K (2009). Establishment of an analytical system for the human fecal microbiota, based on reverse transcription-quantitative PCR targeting of multicopy rRNA molecules. Appl Environ Microbiol.

[13] Fujimoto J, Matsuki T, Sasamoto M, Tomii Y, Watanabe K (2008). Identification and quantification of Lactobacillus casei strain Shirota in human feces with strain-specific primers derived from randomly amplified polymorphic DNA. Int J Food Microbiol.

[14] Fujimoto J, Tanigawa K, Kudo Y, Makino H, Watanabe K (2011). Identification and quantification of viable Bifidobacterium breve strain Yakult in human faeces by using strain-specific primers and propidium monoazide. J Appl Microbiol.

[15] Kikuchi H, Yajima T (1992). Correlation between water-holding capacity of different types of cellulose in vitro and gastrointestinal retention time in vivo of rats. J Sci Food Agr.

[16] Horan TC, Andrus M, Dudeck MA (2008). CDC/NHSN surveillance definition of health care-associated infection and criteria for specific types of infections in the acute care setting. Am J Infect Control.

[17] American Thoracic Society/ Infectious Diseases Society of America (2005). Guidelines for the management of adults with hospital-acquired, ventilator-associated, and healthcare-associated pneumonia. Am J Respir Crit Care Med.

[18] Shimizu K, Ogura H, Goto M, Asahara T, Nomoto K, Morotomi M (2006). Altered gut flora and environment in patients with severe SIRS. J Trauma.

[19] Yamada T, Shimizu K, Ogura H, Asahara T, Nomoto K, Yamakawa K (2015). Rapid and sustained long-term decrease of fecal short-chain fatty acids in critically ill patients with systemic inflammatory response syndrome. J Parenter Enter Nutr.

[20] Bian L, Nagata S, Asahara T, Rahman MS, Ohta T, Yuki N (2011). Effects of the continuous intake of Lactobacillus casei strain Shirota-fermented milk on risk management of long-term inpatients at health service facilities for the elderly. Int J Probiotics Prebiotics.

[21] Kanazawa H, Nagino M, Kamiya S, Komatsu S, Mayumi T, Takagi K (2005). Synbiotics reduce postoperative infectious complications: a randomized controlled trial in biliary cancer patients undergoing hepatectomy. Langenbeck’s Arch Surg.

[22] Lebeer S, Vanderleyden J, De Keersmaecker SC (2010). Host interactions of probiotic bacterial surface molecules: comparison with commensals and pathogens. Nat Rev Microbiol.

[23] Schlee M, Wehkamp J, Altenhoefer A, Oelschlaeger TA, Stange EF, Fellermann K (2007). Induction of human beta-defensin 2 by the probiotic Escherichia coli Nissle 1917 is mediated through flagellin. Infect Immun.

[24] Asahara T, Shimizu K, Nomoto K, Hamabata T, Ozawa A, Takeda Y (2004). Probiotic bifidobacteria protect mice from lethal infection with Shiga toxin-producing Escherichia coli O157:H7. Infect Immun.

[25] Asahara T, Shimizu K, Takada T, Kado S, Yuki N, Morotomi M (2011). Protective effect of Lactobacillus casei strain Shirota against lethal infection with multi-drug resistant Salmonella enterica serovar typhimurium DT104 in mice. J Appl Microbiol.

[26] Selle K, Klaenhammer TR (2013). Genomic and phenotypic evidence for probiotic influences of Lactobacillus gasseri on human health. FEMS Microbiol Rev.

[27] Olivares M, Diaz-Ropero MP, Sierra S, Lara-Villoslada F, Fonolla J, Navas M (2007). Oral intake of Lactobacillus fermentum CECT5716 enhances the effects of influenza vaccination. Nutrition.

[28] Remesy C, Demigne C, Morand C, RJ CJH, Sakata T (1995). Metabolism of short chain fatty acids in the liver. Physiological and clinical aspects of short-chain fatty acids.

[29] Maslowski KM, Vieira AT, Ng A, Kranich J, Sierro F, Yu D (2009). Regulation of inflammatory responses by gut microbiota and chemoattractant receptor GPR43. Nature.

[30] Bleichner G, Blehaut H, Mentec H, Moyse D (1997). Saccharomyces boulardii prevents diarrhea in critically ill tube-fed patients. A multicenter, randomized, double-blind placebo-controlled trial. Intensive Care Med.

[31] Bo L, Li J, Tao T, Bai Y, Ye X, Hotchkiss RS (2014). Probiotics for preventing ventilator-associated pneumonia. Cochrane Database Syst Rev.

[32] Morrow LE, Kollef MH, Casale TB (2010). Probiotic prophylaxis of ventilator-associated pneumonia: a blinded, randomized, controlled trial. Am J Respir Crit Care Med.

[33] Fukuda S, Toh H, Hase K, Oshima K, Nakanishi Y, Yoshimura K (2011). Bifidobacteria can protect from enteropathogenic infection through production of acetate. Nature.

[34] Asahara T, Takahashi A, Yuki N, Kaji R, Takahashi T, Nomoto K (2016). Protective effect of a synbiotic against multidrug-resistant Acinetobacter baumannii in a murine infection model. Antimicrob Agents Chemother.

[35] Knight DJ, Gardiner D, Banks A, Snape SE, Weston VC, Bengmark S (2009). Effect of synbiotic therapy on the incidence of ventilator associated pneumonia in critically ill patients: a randomised, double-blind, placebo-controlled trial. Intensive Care Med.

[36] Asahara T, Takahashi M, Nomoto K, Takayama H, Onoue M, Morotomi M (2003). Assessment of safety of Lactobacillus strains based on resistance to host innate defense mechanisms. Clin Diagn Lab Immunol.

[37] Besselink MG, van Santvoort HC, Buskens E, Boermeester MA, van Goor H, Timmerman HM (2008). Probiotic prophylaxis in predicted severe acute pancreatitis: a randomised, double-blind, placebo-controlled trial. Lancet.

[38] Sheldon T (2010). Dutch probiotics study is criticised for its “design, approval, and conduct”. BMJ.

[39] Gupta VK, Paul S, Dutta C (2017). Geography, ethnicity or subsistence-specific variations in human microbiome composition and diversity. Front Microbiol.

